# Individual opioids, and long- versus short-acting opioids, for chronic noncancer pain: Protocol for a network meta-analysis of randomized controlled trials

**DOI:** 10.1097/MD.0000000000017647

**Published:** 2019-10-25

**Authors:** Atefeh Noori, Jason W. Busse, Behnam Sadeghirad, Reed A. Siemieniuk, Li Wang, Rachel Couban, David N. Juurlink, Lehana Thabane, Gordon H. Guyatt

**Affiliations:** a Department of Health Research Methods, Evidence, and Impact; b Department of Anesthesia,; c The Michael G. DeGroote Institute for Pain Research and Care; d The Michael G. DeGroote Centre for Medicinal Cannabis Research, McMaster University, Hamilton, Ontario, Canada; e Department of Medicine, University of Toronto, Toronto, Ontario, Canada.

**Keywords:** adverse-events, chronic noncancer pain, extended-release, immediate-release, long acting, network meta-analysis, opioids, short acting, systematic review

## Abstract

**Background:**

Opioids are frequently prescribed for the management of patients with chronic non-cancer pain (CNCP). Previous meta-analyses of efficacy and harms have combined treatment effects across all opioids; however, specific opioids, pharmacokinetic properties (ie, long acting vs short acting), or the type of formulation (ie, immediate vs extended release) may be a source of heterogeneity for pooled effects.

**Methods:**

We will conduct a network meta-analysis (NMA) of randomized controlled trials evaluating opioids for CNCP. We will acquire eligible studies through systematic searches of EMBASE, MEDLINE, CINAHL, AMED, PsycINFO, and the Cochrane Central Registry of Controlled Trials (CENTRAL). Eligible studies will have randomly allocated adult CNCP patients to an oral or transdermal opioid versus another type of opioid (or formulation) or placebo, and follow patients for ≥ 4 weeks. We will collect outcome data for pain intensity, physical function, nausea, vomiting, and constipation. Pairs of reviewers will, independently and in duplicate, abstract data from eligible trials and assess risk of bias using a modified Cochrane tool. We will assess coherence of our networks through both a global test, and by comparing direct and indirect evidence for each comparison with node-splitting.

**Results:**

Using a frequentist approach, we will conduct random effects multiple treatment meta-analysis to establish treatment effects of individual opioids for each outcome. The certainty of evidence for pooled treatment effects will be assessed using the Grading of Recommendations, Assessment, Development, and Evaluation (GRADE) approach. We will categorize interventions from most to least effective based on the effect estimates obtained from NMAs and their associated certainty of evidence, as follows: superior to both placebo and alternatives; superior to placebo, but inferior to alternatives; and no better than placebo.

**Conclusion:**

This NMA will determine the relative effectiveness and adverse effects of individual opioids among patients with CNCP. Our results will help inform the appropriateness of assuming similar beneficial and adverse effects of varying opioid formulations.

**Systematic review registration:**

This systematic review is registered with Prospective Register of Systematic Reviews, an international prospective register of systematic reviews (registration no.: CRD42018110331), available at https://www.crd.york.ac.uk/prospero/display_record.php?RecordID=110331.

## Abstract 2

Supplemental Digital Content is available in the text

## 1 Introduction

Chronic noncancer pain (CNCP) is defined as pain, not due to malignancy, that has persisted for at least 3 months.^[1]^ Estimates of the prevalence of chronic pain vary between 15% and 19% among Canadian adults,^[2]^ and opioids are widely prescribed for the treatment of chronic pain, particularly in North America.^[3]^ Despite widespread use, a 2018 systematic review that explored 96 randomized controlled trials of opioids for CNCP found only modest effects for pain and physical function versus placebo; however, heterogeneity of pooled effect estimates was high (*I*^2^ 70% and 64%, respectively) and not explained by subgroup analyses based on: risk of bias, enriched enrollment versus nonenrichment trials, parallel versus cross-over trial design, reported versus converted change scores for treatment effects, and length of follow-up.

Moreover, opioid formulations have been classified based on the onset and duration of action as long acting (LA) or short acting (SA). The pharmacokinetic properties of LA opioids allow for less frequent administration of drug relative to SA opioids, as they provide analgesic effect for 8 to 72 hours (depending on the formulation).^[4]^ There is recommendation regarding the prescription of SA instead of LA opioids for opioid naïve patients with chronic pain.^[5]^

It is possible that some of the heterogeneity in pooled effects of opioids for CNCP may be explained by systematic differences in treatment effect across individual opioids, or by LA and SA profiles. We therefore propose a network meta-analysis (NMA) to explore for differences in treatment effects and harms between individual opioids, and LA versus SA opioids, in patients with CNCP.

## 2 Methods

We registered our protocol on Prospective Register of Systematic Reviews (CRD42018110331) and will adhere to the Preferred Reporting Items for Systematic Review and Meta-analysis for NMA (PRISMA NMA) guidelines (see PRISMA checklist).^[6]^

### 2.1 Search strategy

An academic librarian will develop database-specific search strategies with no language restriction (see supplemental content 1-search result for our proposed search strategy for MEDLINE), and we will systematically search EMBASE, MEDLINE, CINAHL, AMED, PsycINFO, and the Cochrane Central Registry of Controlled Trials (CENTRAL). Reference lists from eligible trials and relevant literature reviews will be scanned for additional trials that may meet our inclusion criteria. No publication status or date limit will be used.

### 2.2 Eligibility criteria

We will include trials that will have randomized patients with CNCP to any currently available oral or transdermal opioid compared to an alternative opioid treatment or placebo, and will have followed participants for at least 4 weeks. We will exclude abstracts, and trial/trial arms with combination products, interventions rarely prescribed in North America or have been taken off the market such as cebranopadol, asimadoline, propoxyphene, or fedotozine.

### 2.3 Study selection

Pairs of reviewers, working independently and in duplicate, will screen titles and abstracts of identified articles and assess the full-text publication for eligibility when one or both reviewers consider a study as potentially eligible. Reviewers will resolve disagreements by consensus and, if disagreements are unresolved, discuss discrepancies with a more experienced team member with relevant expertise. We will pilot this step on 10 randomly selected articles. All screening will be assessed using Rayyan, online systematic review software (https://rayyan.qcri.org/welcome). All eligible articles will be saved in the Endnote X7 library.

### 2.4 Data extraction

Reviewers will extract data independently and in duplicate from eligible studies using standardized forms and a detailed instruction manual. All reviewers will test the data extraction form prior to beginning data abstraction. Our outcomes of interest will be pain intensity, physical function, and adverse events including nausea, vomiting, and constipation. The following information will be abstracted from each study: author, year of publication, baseline characteristics of participants, trial duration, type of intervention and comparison(s), and above-listed outcomes. We will contact study authors if limitations in reporting lead to uncertainties in eligibility, risk of bias, or outcome. If patients provided multiple reports of pain or physical function during follow-up, we will record the last measurement. If pain outcome is available in different measures such as “pain on movement” or “pain at rest” or different time points such as “pain during morning,” “pain mid-day,” or “pain in the evening” we will use “pain at rest” and “pain in the evening.”

### 2.5 Classification of intervention nodes

Regarding pharmacokinetics’ properties, LA opioids are distinguished from SA ones by producing less frequent serum-level fluctuations and releasing the drug more gradually into the bloodstream, thus having a longer half-life (Table 1).^[4,7]^ Different opioid formulations, such as extended release (eg, prolonged release, sustained release, control release, and transdermal forms), prolong the duration that the drug is released into bloodstream, as opposed to immediate release formulations (eg, normal release, or buccal form). We will distinguish between pharmacokinetic properties of opioids (LA or SA opioids) and release formulation (extended release or immediate release) for defining the nodes.

**Table 1:**
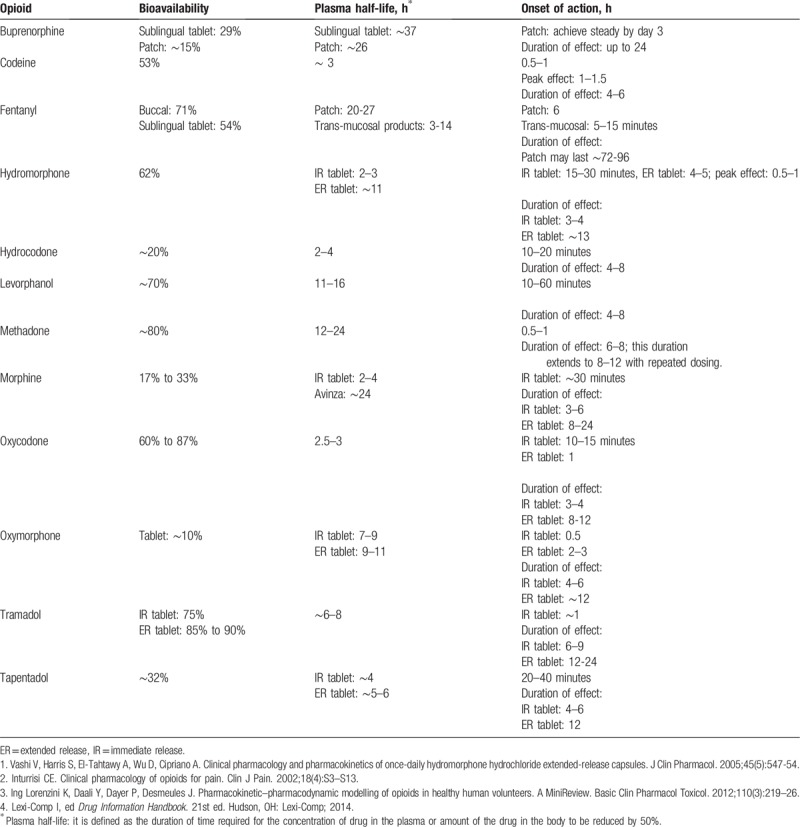
Pharmacokinetics of opioids available for chronic noncancer pain conditions^[1–4]^.

### 2.6 Geometry of the network

The network geometry will be presented to graphically depict the available evidence (each line connecting 2 nodes will indicate a direct comparison between 2 opioids) and may guide readers for the initial interpretation of results.

### 2.7 Risk of bias assessment of individual studies

We will assess the following risk of bias issues in eligible trials: random sequence generation; allocation concealment, blinding of study participants, personnel, and outcome assessors and incomplete outcome data (≥20% missing data will be considered to be at high risk of bias). For this purpose, 2 independent reviewers in duplicate will use a modified Cochrane risk of bias tool for RCTs with the following responses: “definitely or probably yes” (considered as low risk of bias), or “definitely or probably no” (considered as high risk of bias).^[8]^ We will consider allocation concealment adequate if the following methods will have been used: central allocation approaches (“definitely yes”), sequentially numbered drugs with similar appearance, sealed envelopes, opaque, and when patients and investigators will have been blinded (“probably yes”).^[8]^ Discrepancies in assessment of risk of bias will be resolved by discussion, or third party adjudication if needed.

### 2.8 Data synthesis and statistical methods

For each direct comparison, we will calculate the weighted mean difference and the associated 95% confidence intervals (CIs) for continuous outcomes. For dichotomous outcomes, we will calculate odds ratio with corresponding 95% CIs. We will use the methods described in the Cochrane Handbook^[9]^ to impute the mean and standard deviation (SD) when median, range, and sample size are reported, and to impute the SD if the standard error or SD for the differences are not reported. If pain or physical functioning was measured by different instruments, we will abstract the most commonly reported scale, for example, 0 to 10 numerical rating scale or visual analogue scale for pain; and SF-36 physical component summary score, physical functioning subdomain, or WOMAC function subscale for physical functioning. We will use change scores from baseline to end of follow-up rather than end-of-study scores, in order to account for inter-patient variability. We will calculate change score for studies that do not report them using the baseline and end-of-study score and a correlation coefficient derived from the largest trial at lowest risk of bias. We will perform pairwise meta-analysis of the available direct comparisons using the DerSimonian–Laird random-effects model for all outcomes.

We will use the network estimate of treatment effects for continuous outcomes to calculate the risk difference for achieving the minimally important difference; the smallest change in a patient-reported outcome that patients perceive as important is one minimally important difference. We will perform NMA to synthesize the available evidence from the entire network of trials by integrating direct and indirect estimates for each comparison into a single summary treatment effect. We will use a frequentist random-effects model using the methodology of multivariate meta-analysis to assess the comparative effectiveness of eligible interventions.

### 2.9 Assessment of inconsistency

We will identify issues of incoherence (direct and indirect effect estimates are not similar) by comparing direct evidence (ie, estimates from pairwise comparisons) with indirect evidence (ie, estimates from NMA) using the node splitting method.^[10]^ In this approach, incoherence is assessed locally by statistical evaluation of the difference between direct and indirect estimates for a specific comparison in the loop. We will assume a common heterogeneity estimate across the network. In case of incoherence in a closed loop of evidence, the certainty of evidence of each estimate can lead us to decide which estimate to believe.^[11]^ We will also address the coherence assumption in the entire network using “design-by-treatment” model as described by Higgins et al.^[12]^ In case we find significant incoherence in the network (highly significant *P* value from design-by-treatment model), we will perform NMA using inconsistency model. If we have at least 10 studies, we will construct a contour enhanced funnel plot for each treatment comparison to assess for small-study effects. To assess the funnel plot asymmetry we will use Harbord et al^[13]^ rank correlation and Egger et al test^[14]^ as well as visual inspection. We will use Stata (StataCorp, Release 15.1, College Station, TX) for all analysis. All comparisons will be 2 tailed using a threshold *P* ≤ 0.05.

### 2.10 Ranking of competing opioids

We will estimate ranking probabilities using the surface under the cumulative ranking curve (SUCRA: values range from 0 “the worst possible SUCRA” to 100 “the best possible”). We will also use a novel approach in which we will classify opioids first based on their effectiveness versus placebo and then versus other competing interventions and finally according to Grading of Recommendations, Assessment, Development, and Evaluation (GRADE) certainty of evidence ratings. Opioids will be sorted into 3 groups: among the most effective (superior to both placebo and to at least 1 intervention superior to placebo or no treatment); superior to placebo, but not superior to any other intervention; or no more effective than placebo. The standard for superiority will be excluding a relative effect of 1.0. For harms, the categorization will be least harmful (no different from placebo); more harmful than placebo but no more harmful than any other intervention; and more harmful than at least 1 other intervention. Within each group of 3 categories we will separate those interventions with moderate or high certainty evidence from those with only low or very low certainty evidence relative to placebo.

### 2.11 Additional analysis

We will use the Q statistic and *I*^2^ to determine statistical heterogeneity for direct meta-analysis. We will assess the impact of studies with shorter duration of follow-up, higher risk of bias, and enriched study design by removing them from the pairwise meta-analysis. If the produced results will not be robust with the results obtained from primary model, we will remove them from further analysis. We will also, conduct network meta-regression assuming a common fixed coefficient across comparisons to explore the effect of opioids formulation (extended vs immediate release) on all outcomes, if we will have enough studies.

### 2.12 Certainty of the evidence

We will use the GRADE approach^[15]^ to evaluate the certainty of evidence on an outcome-by-outcome basis and classify evidence as high, moderate, low, or very low certainty based on the limitations in risk of bias, imprecision, inconsistency, indirectness, and publication bias. The GRADE approach also will be used to assess the certainty of evidence from indirect and network (mixed) effect estimates in duplicate and independently. We will visually examine the network map to find the dominant lowest-order loop^[16]^ available for indirect comparisons; the certainty of evidence will be the lower of the ratings for the informing direct estimates contributing to the loop of evidence.^[17]^ In the GRADE approach for NMA, indirect effect estimates may be further rated down for intransitivity (the transitivity assumption implies to the similarity of trials in population, intervention, comparison, and trial methodology informing the indirect comparison in a closed-loop of evidence). When the certainty of the direct evidence is judged to be high and the contribution of it to the network estimate is at least as great as that of the indirect evidence, the certainty rating of network estimate will be only based on the direct evidence.^[11]^ When there is no indirect evidence, the certainty of network estimate will be graded according to the direct evidence.

## 3 Discussion

The results of our proposed study will provide the comparative effectiveness of individual opioids for the treatment of CNCP population. This is common that in systematic review individual opioids or different formulations of opioids (extended or immediate release) have pooled, assuming similar effect size; this NMA will inform whether this historical practice of pooling across individual opioids is a source of heterogeneity or not.

Our planned review has several strengths including a comprehensive search of published and unpublished results; comparing all individual opioids in terms of the benefits and harms; and our study will use an innovative approach for sorting opioids to provide a clear guide for action for health care providers. However, there might be some challenges for the current review as well. For instance, if the number of included studies will be inadequate, the ability to explore the source of anticipated inconsistencies would be restricted.

For knowledge translation purpose, we will publish our results in an accessible peer-reviewed journal and present our findings at international and national scientific conferences.

## Author contributions

**Conceptualize and design of the study:** Atefeh Noori, Jason W. Busse, Gordon H. Guyatt.

**Designed systematic search strategy:** Rachel Couban.

**Drafted the manuscript:** Atefeh Noori.

**Provided methodological advice:** Reed A. Siemieniuk, Li Wang.

**Revised the manuscript:** Jason W. Busse, Behnam Sadeghirad, Li Wang, Reed A. Siemieniuk, Rachel Couban, David Juurlink, Lehana Thabane, Gordon H. Guyatt.

All authors reviewed, provided critical feedback, and approved this protocol.

## Supplementary Material

Supplemental Digital Content
