# Supplementary material for: Individual opioids, and long- versus short-acting opioids, for chronic noncancer pain: Protocol for a network meta-analysis of randomized controlled trials
Source: Medicine (Baltimore). 2019 Oct 25;98(43):e17647. doi: 10.1097/MD.0000000000017647 (PMC6824796; doi:10.1097/MD.0000000000017647)
Supplement: Supplemental Digital Content [file medi-98-e17647-s001.doc]

**Supplemental content 1: Literature search strategy**

**Database: OVID Medline Epub Ahead of Print, In-Process & Other Non-Indexed Citations, Ovid MEDLINE(R) Daily and Ovid MEDLINE(R)**

**----------------------------------------------------------------------------------------------**

1. exp Analgesics, Opioid/

2. opioid$.mp.

3. (Asimadoline or Alvimopam or Fedotzine or Fentanyl).mp.

4. Hydrocodone.mp.

5. (Hydromorphone or Levorphanol or Meperidine or

Morphine).mp.

6. (Oxycodone or Oxymorphone or Pentazocine or Propoxyphene).mp.

7. (Sufentanil or Tramadol).mp.

8. exp Codeine/

9. Codeine.mp.

10. or/1-9

11. exp Morphine/

12. morphine.tw.

13. morphia.mp.

14. ms contin.rn,mp.

15. oramorph sr.mp.

16. duramorph.mp.

17. 57-27-2.rn.

18. morphinene.mp.

19. anpec.mp.

20. duromorph.mp.

21. epimorph.mp.

22. miro.mp.

23. morfin.mp.

24. morfine.mp.

25. morphin.mp.

26. morphinium.mp.

27. morphium.mp.

28. opso.mp.

29. skenan.mp.

30. trama.mp.

31. (n-methylmorphine or n methylmorphine or isocodeine or ardinex).mp.

32. (phentanyl or fentanest or sublimaze or fentora).mp.

33. (duragesic or durogesic).mp.

34. (hydrocodon or dihydrocodeinone or dicodid or robidone or hydrocodeinonebitartrate or hydrocon).mp.

35. (dihydromorphinone or hydromorphon or palladone or laudacon or dilaudid).mp.

36. (codinovo or hycodan or hycon).mp.

37. (dihydromorphinone or hydromorphon or palladone or laudacon or dilaudid).mp.

38. (levodroman or levorphan or levo-dromoran or levodromoran).mp.

39. l dromoran.mp.

40. (pethidine or isonipecain or dolsin or dolosal or dolin or dolantin).mp.

41. (dolargan or lidol or lydol or Demerol or dolcontral).mp.

42. (dihydrohydroxycodeinone or oxycodeinon or dinarkon or eucodal).mp.

43. (hydroxycodeinon or oxiconum or oxycone or oxycontin).mp.

44. (pancodine or theocodin or dihydrone).mp.

45. (numorphan or talwin or lexir or fortral).mp.

46. (sulfentanyl or sulfentanil or sufenta).mp.

47. (tramadolhameln or tramadolor or tramadura or tramagetic or tramagit).mp.

48. (tramake or tramal or tramex or tramundin or trasedal).mp.

49. (ultram or zamudol or zumalgic or zydol or zytram).mp.

50. (adolonta or contramal or amadol or biodalgic or jutadol or nobligan or prontofort or takadol).mp.

51. (theradol or tiral or topalgic or tradol or tradolpuren or tradonal or tralgiol).mp.

52. (tramadorsch or biokanol or tramadin or tramadoc). mp.

53. exp narcotics/

54. or/11-53

55. 10 or 54

56. (chronic adj6 pain$).mp.

57. Chronic Disease/

58. exp Pain/

59. Low back pain.mp. or exp Back Pain/

60. backache$.mp.

61. Fibromyalgia.mp. or exp Fibromyalgia/

62. exp Whiplash Injuries/ or Whiplash.mp.

63. Irritable bowel syndrome.mp. or exp Irritable Bowel Syndrome/

64. Irritable colon.mp.

65. Temporomandibular joint syndrome.mp. or exp

Temporomandibular Joint Dysfunction Syndrome/

66. Tension headache$.mp. or exp Tension-Type Headache/

67. Headache/

68. exp Cumulative Trauma Disorders/ or Repetitive strain syndrome.mp.

69. Osteoarthritis.mp. or exp Osteoarthritis/

70. Rheumatoid arthritis.mp. or exp Arthritis, Rheumatoid/

71. exp Diabetic Neuropathies/

72. diabetic neuropath$.mp.

73. Post herpetic neuralgia.mp. or exp Neuralgia, Postherpetic/

74. Postherpetic neuralgia.mp.

75. exp Phantom Limb/ or Phantom limb pain.mp.

76. exp Brachial Plexus Neuritis/ or cervicobrachial pain syndrome.mp.

77. globus syndrome.mp.

78. exp Headache Disorders/

79. neuropathic pain$.mp.

80. neuralgia.mp. or exp Neuralgia/

81. Pain Measurement/

82. diabetic neuropath$.mp.

83. polyneuropathies.mp. or exp Polyneuropathies/

84. polyneuropathy.mp.

85. or/56-84

86. randomized controlled trial$.mp.

87. randomized controlled trial.pt.

88. random allocation/

89. double-blind method/

90. single-blind method/

91. randomi?ed controlled trial$.mp.

92. controlled clinical trial.pt.

93. randomized controlled trial.pt.

94. ((singl$ or double$ or trebl$ or tripl$) adj25 (blind$ or mask$)).mp.

95. random$.mp.

96. placebo$.mp.

97. cross-over studies.sh.

98. latin square:.tw.

99. clinical trial.pt.

100. exp evaluation studies/

101. Retrospective Studies/ or follow up studies/ or prospective studies/

102. or/86-101

103. animals/ not humans/

104. 102 not 103

105. 55 and 85 and 104

106. 105 not ((acute or postoperative).ti,ab. not chronic. mp.)

107. 55 and 85 and comparative study/ and chronic.mp.

108. 107 not 103

109. 108 not ((acute or postoperative).ti,ab. not chronic. mp.)

110. 109 or 106

111. 110 not (exp neoplasms/ not chronic.mp.)
